# Supplementary material for: MetaRibo-Seq measures translation in microbiomes
Source: Nat Commun. 2020 Jun 29;11:3268. doi: 10.1038/s41467-020-17081-z (PMC7324362; doi:10.1038/s41467-020-17081-z)
Supplement: Supplementary file 10 — Supplementary Data 7 [file 41467_2020_17081_MOESM10_ESM.zip › File2/Confidence_High_Taxonomy/200519_out.krona.html]

Javascript must be enabled to view this page.

members
magnitude
magnitudeUnassigned
count
unassigned
taxon
rank

200519\_out

5

superkingdom
5
2

phylum
5
1239

5
class
186801

186802
order
5

1897047

SRS011405\_contig\_number\_17665SRS024009\_contig\_number\_contig-100\_5473.5473SRS143876\_contig\_number\_36044SRS893172\_contig\_number\_6682SRS893187\_contig\_number\_7765
5
species
